# Habitat Dynamics and Protected Area Effectiveness of the Endangered *Paphiopedilum* Subgen. *Brachypetalum* (Orchidaceae) Under Climate Change

**DOI:** 10.1002/ece3.73529

**Published:** 2026-04-20

**Authors:** Hao Zhang, Mingtai An, Jianghong Yu

**Affiliations:** ^1^ College of Forestry Guizhou University Guiyang China; ^2^ College of Life Sciences Guizhou University Guiyang China

**Keywords:** BIOMOD2, climate change, spatial pattern, subgen. *Brachypetalum*

## Abstract

*Paphiopedilum*, owing to its high ornamental value and ecological sensitivity, has become one of the flagship groups for global biodiversity conservation. Our study focuses on *Paphiopedilum* subgen. *Brachypetalum*, employing the BIOMOD2 package to build ensemble models (EMmedian) that predict the responses and shifts of suitable habitats under LIG, MH, current, and future (2050s and 2090s under SSP scenarios) conditions, and overlaying these predictions with existing protected areas to assess their effectiveness for current and future conservation. The results showed that: (1) the ensemble models exhibited high predictive performance (AUC > 0.9 and TSS > 0.8), and mean temperature of the driest quarter (bio9) and precipitation of the driest month (bio14) are the key driving factors influencing the distribution of subgen. *Brachypetalum*. (2) At present, subgen. *Brachypetalum* occurs mainly in three core regions: the southern edge of the Yunnan–Guizhou Plateau adjoining northern Indochina, the Guizhou–Guangxi border, and the Yunnan–Guizhou junction zone. In future climate scenarios, the suitable range of subgen. *Brachypetalum* is projected to shrink (the area of highly suitable habitat projected to decrease by 75.85%–99.92%) and migrate northward to northwestward, centering on southwestern Guizhou Province. (3) Although existing protected areas provide partial protection for subgen. *Brachypetalum*, they are inadequate to fulfill conservation needs under future climate conditions. To cope with global climate change, we recommend establishing or expanding reserves or conservation sites in stable and highly suitable areas within the border region of Yunnan, Guizhou, and Guangxi, centered on southwestern Guizhou, to ensure the long‐term persistence of subgen. *Brachypetalum* under changing climatic conditions.

## Graphical abstract

This study explores how global climate change reshapes the suitable habitats and conservation potential of *Paphiopedilum* subgen. *Brachypetalum*, a flagship group of endangered terrestrial orchids in East and Southeast Asia. Using 580 field‐verified and 75 global occurrence records, we applied an ensemble modeling framework (BIOMOD2) integrating 11 algorithms to reconstruct past, present, and future habitat suitability under multiple climate scenarios. The results reveal pronounced temporal dynamics: “LIG extreme contraction – MH significant expansion – Current peak – Future decline,” and a consistent north–northwestward shift toward southwestern Guizhou, identifying this region and the Yunnan–Guizhou–Guangxi border as key priorities for adaptive conservation.

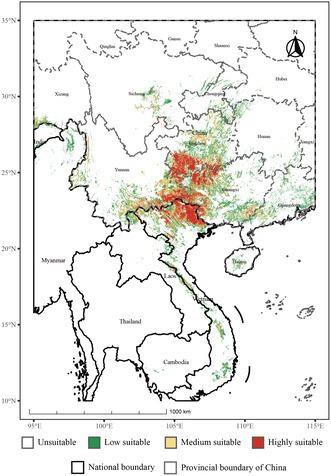

## 1 Introduction

*Paphiopedilum* is one of the most typical, primitive, and rare terrestrial genera within the Orchidaceae family, with great research significance, and is recognized as a flagship species for biodiversity conservation (Cai et al. 2024; Ye et al. 2025). Moreover, the unique floral structure and vibrant coloration of *Paphiopedilum*, with its slipper‐like labellum resembling a fairy's shoe, grant it the name “slipper orchid” and make it highly prized for ornamental purposes (Jiang et al. 2025). Nevertheless, the biological constraints of *Paphiopedilum*, combined with habitat loss from environmental changes and persistent pressures from illegal harvesting and habitat degradation, have placed several wild populations at imminent risk of extinction (Wang et al. 2021). At present, all *Paphiopedilum* species are included in Appendix I of the Convention on International Trade in Endangered Species of Wild Fauna and Flora (CITES) and in China's List of National Key Protected Wild Plants—version 2021. Although the conservation status has improved for some species through in situ and ex situ protection, the overall conservation system remains incomplete and requires further strengthening (Smith et al. 2011; Zhou et al. 2021; Ye et al. 2023). According to the latest World Checklist of Vascular Plants (WCVP, Version 12), there are approximately 109 species of *Paphiopedilum* worldwide, and mainstream studies classify them into three subgenera (*Paphiopedilum*, *Brachypetalum*, and *Parvisepalum*) (Górniak et al. 2025; Yuan et al. 2010). The natural range of subgen. *Brachypetalum* extends broadly, from central‐southern Vietnam in the south to northern Guizhou, China in the north, westward to the Himalayan Mountains, and eastward to the coastal regions of the South China Sea, but its actual populations occupy very limited areas and grow in clusters, showing an aggregated distribution pattern typical of widely distributed but rare species (Zhang et al. 2022). Existing research on subgen. *Brachypetalum* has primarily emphasized phylogenetic relationships and genetic diversity (Górniak et al. 2025; Sun et al. 2022), resource distribution and conservation (Zhang et al. 2022), and horticultural practices (Zeng et al. 2016), yet comprehensive studies on its climate change responses and adaptive conservation strategies are still insufficient.

The IPCC assessment reports indicate that the global mean surface temperature is projected to rise inevitably by 0.3°C–4.8°C during the 21st century, accompanied by substantial changes in precipitation patterns (Thackeray et al. 2022). Climate change has altered the spatiotemporal patterns of global biodiversity, exerting particularly strong impacts on species with narrow distribution ranges and sensitive ecological niches (Janni et al. 2024; Alexander et al. 2018; Parmesan 2006). For plant species, continuous warming may force them to migrate toward higher latitudes or elevations (Wang, Li, et al. 2025; Randin et al. 2009), while altered humidity patterns undermine their reliance on stable water availability (Kreuzwieser and Gessler 2010; Brouder and Volenec 2008). Climate warming and altered precipitation patterns not only directly reshape species’ suitable ranges but indirectly alter species’ long‐term survival prospects by influencing individual physiological processes, population recruitment, and community assembly mechanisms (Shi et al. 2023). Increasing evidence suggests that extreme heat events and uneven precipitation patterns have become key factors limiting the distribution of many endangered plant species. Rising temperatures and shifting precipitation patterns have been confirmed as the primary environmental drivers determining changes in plant habitats (Trew and Maclean 2021; McLaughlin et al. 2017). The subgen. *Brachypetalum* itself grows within plant communities in karst regions characterized by shallow soils or fragmented terrain. Unstable hydrothermal conditions will further exacerbate the vulnerability of species distribution ranges, leading to shifts in species distribution and habitat fragmentation.

Species distribution models (SDMs) are ecological models capable of quantifying the impact of climate change on biogeographic patterns and predicting shifts in suitable habitats. Against the backdrop of accelerating global climate change and growing awareness of biodiversity conservation, SDMs have undergone rapid development, providing a vital tool for assessing plant responses to climate change. Currently, SDMs are widely applied, such as in biodiversity conservation (Li et al. 2024), invasive species prevention (Wang, Wu, et al. 2025), phylogeographic and evolutionary reconstruction (Wan et al. 2024), ecosystem service evaluation (Baldan et al. 2024), and disease transmission control (Thumsová et al. 2025). By employing SDMs, researchers can reveal how climate variables shape species niches, evaluate potential migration pathways under different climate scenarios, and provide scientific guidance for large‐scale conservation and management strategies (Hellegers et al. 2025; Zhao et al. 2023). Within SDMs, ecological niche models (ENMs) integrate species distribution records with environmental variables to predict both current suitable habitats and reconstruct distribution patterns across different time periods, thereby revealing the dynamic evolution of distribution patterns (Elith et al. 2011; Forester et al. 2013; Peterson and Vieglais 2001). However, ENM outputs are sensitive to algorithmic choices, which can limit the reliability of single‐model outputs in representing real biological responses to climatic change.

To address this limitation, we used the BIOMOD2 package to predict the potential distribution areas of subgen. *Brachypetalum* for nine climate scenarios using 11 individual models and three ensemble methods (Thuiller et al. 2019). The prediction results were integrated with spatial data on protected areas to assess the effectiveness of the existing conservation network. We hypothesize that climate change will significantly alter the spatial distribution of suitable habitats for subgen. *Brachypetalum* and may lead to mismatches between future suitable areas and the current protected area network. This study aims to address the following questions: (A) Which environmental variables most strongly determine the distribution of subgen. *Brachypetalum*? (B) How do the patterns and dynamics of its suitable habitats vary across historical, current, and projected future climates? (C) What conservation potential do existing protected areas hold for subgen. *Brachypetalum* in the context of climate change? Which areas should be prioritized for future conservation?

## 2 Materials and Methods

### 2.1 Study Species and Area

**FIGURE 1:**
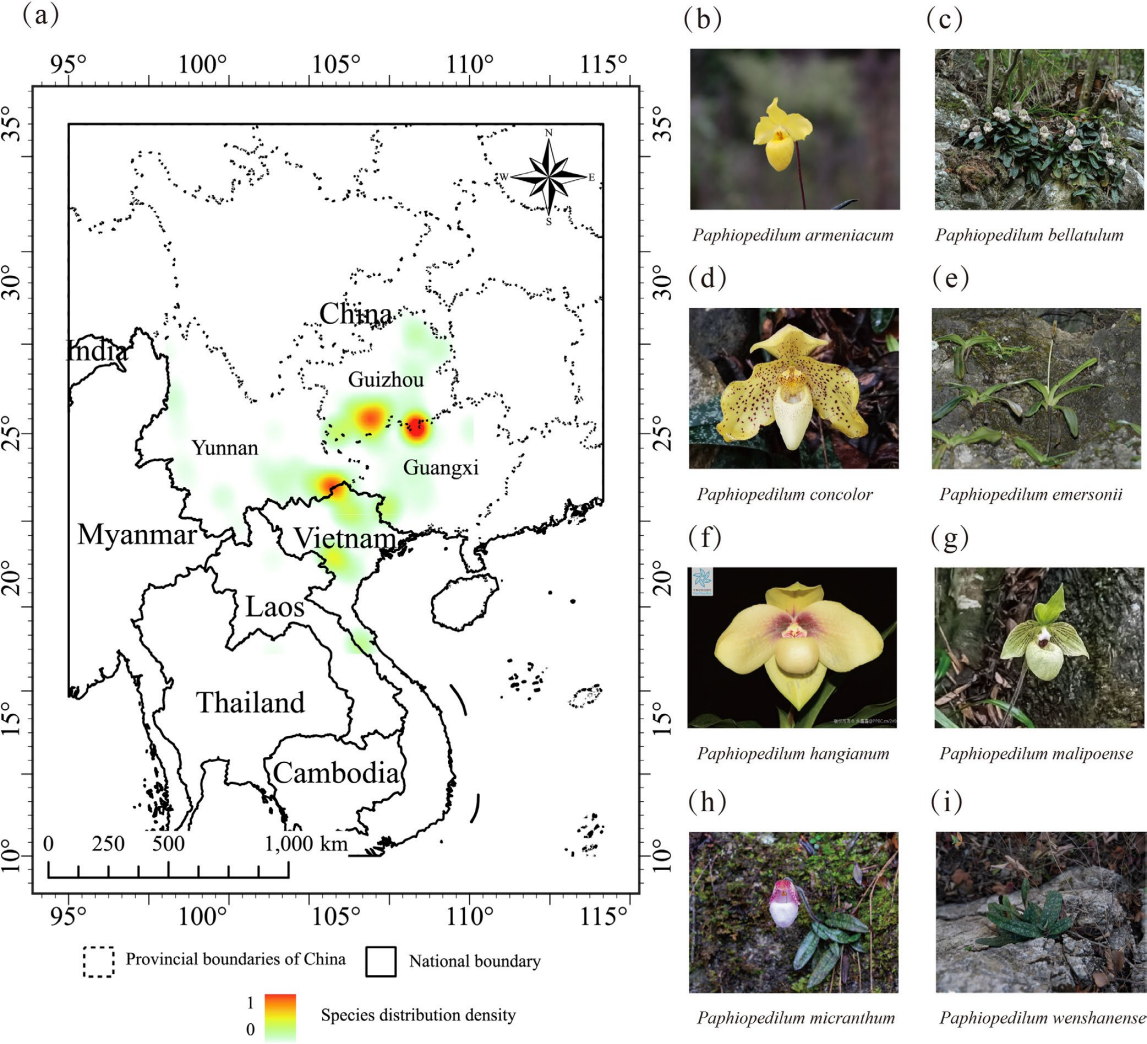
Study area and species. (a) Study area and kernel density distribution of occurrence records, with kernel density probability ranging from 0 to 1. (b–i) Field photographs of eight species of subgen. *Brachypetalum*. f was downloaded from the Plant Photo Bank of China (PPBC), credited to Xinxin Zhu; the others were taken by the research group during field surveys.

At present, subgen. *Brachypetalum* comprises 12 recorded species, mainly distributed in southwestern China and neighboring regions of Southeast Asia. Due to limited accurate distribution data, the core dataset for this study was derived from field surveys. We selected eight species with confirmed occurrences in China as modeling objects (Figure 1b–i) and integrated distribution point data from neighboring countries to supplement ecological niche information. These selected species encompass all representative taxa of the subgenus found in China, and by integrating cross‐border occurrence data, the study effectively reflects the overall distribution pattern of subgen. *Brachypetalum* in the East Asia–Southeast Asia region (Wu 2009; Liu et al. 2009). Regarding the study area, to ensure complete coverage of potential suitable habitats and minimize boundary effects, the modeling extent was extended to 95° E–115° E and 10° N–35° N based on occurrence data (Figure 1a) (Barve et al. 2011).

The study area is situated within the East Asian monsoon region and the northern margins of tropical Southeast Asia, and is characterized by complex topography and diverse climatic conditions. The landscape is predominantly composed of mountainous and karst terrains, with warm and humid summers, and relatively cool and dry winters. Annual precipitation generally ranges from 500 to 2000 mm, exhibiting pronounced seasonal variability (Chen et al. 2024). These environmental gradients contribute to high habitat heterogeneity and support rich biodiversity, particularly for specialized and range‐restricted taxa such as subgen. *Brachypetalum*. In terms of conservation status, the region encompasses several national nature reserves and recognized biodiversity hotspots.

### 2.2 Species Occurrence Data

The species distribution data were primarily obtained from 580 recent field investigations conducted by our research team, including (i) targeted field surveys across China from June 2019 to the end of 2020, (ii) ongoing orchid monitoring in Guizhou Province since May 2024, and (iii) long‐term field records accumulated by our group, combined with (iv) 75 high‐quality global occurrence records downloaded from the GBIF database (GBIF 2025), resulting in a total of 655 distribution points.

During data processing, we manually filtered out three types of outliers: those exceeding predetermined distribution boundaries, those with low spatial accuracy, and those significantly deviating from overall ecological niche conditions. Subsequently, spatial thinning was applied to all occurrence points using the R package spThin. This approach mitigated sampling bias, reduced the risk of model overfitting, and significantly improved computational efficiency during the model calibration phase (Aiello‐Lammens et al. 2015). The minimum distance was set to 1 km, and 100 thinning iterations were performed; the dataset with the best thinning performance was selected, resulting in 188 valid occurrence points.

As *Paphiopedilum* subgen. *Brachypetalum* is under China's national protection list, all coordinates were anonymized to prevent illegal harvesting, and individual occurrence locations are not displayed on the maps, only the overall regional distribution pattern is illustrated (Figure 1a).

### 2.3 Environmental Variables

The environmental variables included climatic, topographic, and edaphic factors (Table 1). Climatic and topographic variables were obtained from the WorldClim2.1 database (https://www.worldclim.org/) (Fick and Hijmans 2017), with Slope and Aspect derived from elevation. Soil variables were extracted from the D1 soil layer of the HWSD2.0 database (https://www.fao.org/soils‐portal/data‐hub/soil‐maps‐and‐databases/harmonized‐world‐soil‐database‐v20/en/) (Sinitambirivoutin et al. 2024). Climatic datasets included multiple temporal periods: Last Interglacial (LIG), Mid‐Holocene (MH), Current, and future projections (2050 and 2090) based on the BCC‐CSM2‐MR model, which has been shown to perform well in simulating climate patterns over East Asia (Shuping et al. 2023; Guo et al. 2022). Future projections were conducted under SSP1–2.6, SSP2–4.5, and SSP5–8.5 scenarios.

**TABLE 1:** Description and ranges of the 10 environmental variables in modeling.

Code	Environmental variable	Unit	VIF	Min	Mean	Max
bio2	Mean Diurnal Range	°C	3.05	6.26	7.98	12.68
bio5	Max Temperature of Warmest Month	°C	1.72	24.20	28.67	35.10
bio9	Mean Temperature of Driest Quarter	°C	1.70	4.78	11.16	22.78
bio13	Precipitation of Wettest Month	mm	2.81	183.00	277.00	509.00
bio14	Precipitation of Driest Month	mm	4.47	3.00	19.23	55.00
Aspect	Aspect	°	1.09	0.22	170.64	359.89
Slope	Slope	°	1.29	0.09	4.42	22.79
BSAT	Base saturation	%	1.64	1.00	37.15	83.00
COARSE	Soil coarse fragment content	%	1.62	1.00	20.49	36.00
SAND	Sand content	%	2.35	1.00	21.94	72.00

*Note:* The number retains two decimal places.

Because of the difference in the unit of WorldClim database, temperature variables in paleoclimate datasets were numerically normalized (divided by 1000) to ensure consistency with other climate scenarios. All variables were standardized to a 30 arc‐sec spatial resolution using bilinear interpolation and carefully aligned in spatial extent and projection.

To minimize multicollinearity, the usdm (2.1–7) R package was applied to compute the variance inflation factor (VIF) for each candidate variable, keeping variables with VIF ≤ 10 (Naimi and Araújo 2016), followed by Pearson correlation analysis to exclude variables with an absolute correlation coefficient ≥ 0.8. Based on this, we prioritized variables with clear ecological relevance according to the adaptive characteristics of subgen. *Brachypetalum*. A total of 10 environmental variables were retained for modeling: 5 climatic (bio2, bio5, bio9, bio13, bio14), 2 topographic (Slope, Aspect), and 3 edaphic (BSAT, COARSE, SAND).

### 2.4 Model Construction and Evaluation

We used the BIOMOD2 (v4.2–6‐2) R package to construct ensemble models, integrating 11 commonly used algorithms (MAXENT, ANN, CTA, FDA, GAM, GBM, GLM, MARS, RF, SRE, and XGBOOST) to simulate the potential geographic distribution of subgen. *Brachypetalum*. First, when generating pseudo‐absence points, we adopted the disk strategy, which aligns with the island‐like distribution characteristics of subgen. *Brachypetalum* to avoid model bias caused by inappropriate background point selection. A 10 km exclusion radius was set around each presence point, and an equal number of pseudo‐absence points (188 pseudo‐absence points) were randomly generated to balance the presence/absence ratio and reduce spatial autocorrelation (Jinga and Manyangadze 2024). Second, the model parameters were configured using the “bigboss” optimization strategy, which automatically adjusts algorithm‐specific parameter settings to enhance comparability and robustness among the different modeling algorithms. For example, the RF model used 500 trees with mtry = 2 and node size = 5. The GBM model was configured with 2500 trees, an interaction depth of 7, a learning rate of 0.001, and threefold internal cross‐validation. The MAXENT model used multiple feature classes (linear, quadratic, product, hinge, and threshold) with a regularization multiplier of 1. For cross‐validation, we employed k‐fold (*k* = 5) to ensure each existing data point could be utilized for prediction and evaluation, with five repetitions. Ultimately, each algorithm ran 25 times, yielding a total of 275 distinct model simulations across all algorithms.

When evaluating models, we employ two metrics: the true skill statistic (TSS) and the area under the receiver operating characteristic curve (AUC). AUC is a probabilistic indicator derived from the ROC curve that reflects the model's ability to distinguish between presence and absence points across different thresholds. Its values range from 0 to 1, with values closer to 1 indicating stronger discriminatory power. An AUC ≥ 0.9 indicates excellent predictive performance (Jiménez‐Valverde 2012). When high‐quality species distribution data are available, TSS retains the advantages of the Kappa statistic while accounting for the optimal balance between specificity and sensitivity thresholds (Leroy et al. 2018). TSS is calculated by combining sensitivity and specificity, defined as TSS = sensitivity + specificity−1. A TSS ≥ 0.8 indicates excellent model performance (Allouche et al. 2006). Therefore, we consider models with TSS ≥ 0.8 and AUC ≥ 0.9 as high‐performance models and select them for ensemble modeling.

During ensemble construction, three ensemble strategies in BIOMOD2 (EMmean, EMwmean, and EMmedian) were tested using the TSS as the weighting criterion, and the optimal ensemble model was selected to evaluate the relative importance of environmental variables (Guo et al. 2025; Chen et al. 2025).

### 2.5 Spatial Analysis and Statistics

During habitat suitability prediction, the selected high‐performing single models were first projected, and their results were integrated with the optimal ensemble model to produce the final projection. Because the ensemble model produced a continuous probability surface (0–1000), we reclassified it using the terra R package into binary suitable/unsuitable categories, where cells with values > 600 were defined as suitable habitats. Suitable habitats were classified into three levels of suitability, 600–700 (low), 700–800 (medium), and > 800 (high), to more precisely characterize the spatial distribution pattern and habitat quality gradient of subgen. *Brachypetalum*. This threshold was determined based on commonly used thresholds in similar studies, combined with the observed distribution patterns under current conditions and species‐specific ecological characteristics, ensuring consistent classification across scenarios (Liu et al. 2024; Wan et al. 2024; Thuiller et al. 2019).

Based on the ensemble model projections, we performed a multidimensional spatial analysis of the distributional dynamics of subgen. *Brachypetalum*. First, the area of suitable habitats was calculated for different time periods (LIG, MH, Current, 2050, and 2090) and climate scenarios (SSP1–2.6, SSP2–4.5, SSP5–8.5). Second, using the BIOMOD_RangeSize() function, we compared the total area and high‐suitability area of the current distribution with those of the binary habitat raster under various projected scenarios. This allowed us to identify and quantify three types of habitat change, stable areas, loss areas, and gain areas, thereby characterizing the spatial processes of range contraction and expansion. Finally, based on the spatial analysis results, we further calculated the center‐of‐mass shifts of the total suitable area and highly suitable area. This enabled us to infer potential migration trajectories, assess spatial stability and refuge persistence under climate change, and provide quantitative evidence for delineating future conservation priority areas.

### 2.6 MESS and MoD Analyses

To quantify uncertainties associated with environmental extrapolation in future climate projections, we employed the dismo (v1.3–16) R package to calculate both the multivariate environmental similarity surface (MESS) and the most dissimilar variable (MoD) (Frans et al. 2022; Radomski et al. 2022). The MESS analysis quantifies the degree of similarity between environmental conditions in projected areas and those within the model's training environment, producing a continuous raster of environmental similarity values. Negative values indicate regions where environmental conditions exceed the training range, implying extrapolation uncertainty; a value of 0 represents an identical environment, while values between 1 and 100 reflect the degree of environmental variation. The MoD analysis identifies the environmental variable most responsible for these dissimilarities, generating a categorical raster in which each value corresponds to a specific variable contributing to extrapolation differences (Lv et al. 2025). MoD helps explain the sources of prediction uncertainty and identifies which environmental variables exhibit the most pronounced changes. By combining MESS and MoD analyses, we can not only spatially identify regions with extrapolation risk but also determine the specific environmental drivers, thereby providing contextual information for interpreting model results.

### 2.7 Protected Area Coverage and Gaps

To evaluate how well the current protected area network covers the distribution of subgen. *Brachypetalum*, multiple protected area datasets were compiled and integrated. Data for protected areas within China were retrieved from Zenodo (NSII 2024), and data for areas beyond China were taken from the World Database on Protected Areas (WDPA) (2025) (www.protectedplanet.net). During data integration, Chinese protected area vector data were used to replace the China portion of the WDPA dataset, and the merged dataset was resampled to a uniform spatial resolution of 30 arc‐sec. By overlaying current and future highly suitable habitat areas, as well as areas of gain, stability, and loss, with protected area boundaries, we calculated conservation effectiveness indicators: (i) the protected area coverage of current highly suitable habitats; (ii) the protected area coverage of future highly suitable habitats; and (iii) the stable, gained, and lost areas of highly suitable habitats within protected areas across different time periods.

All data processing, analysis, and mapping were conducted in R 4.3.2 and ArcGIS Pro 3.3.1, with raster area calculations performed using the cellSize() function of the terra R package under the WGS84 geographic coordinate system. The administrative boundary basemap was obtained from the National Platform for Common Geospatial Information Service of China (http://bzdt.ch.mnr.gov.cn/index.html), with the review approval number GS (2020) 4619.

## 3 Results

### 3.1 Model Evaluation

Across 275 independent single‐model runs, most algorithms exhibited strong predictive ability, and 75 single models satisfied the selection thresholds (TSS ≥ 0.8 and AUC ≥ 0.9). Among all algorithms, RF demonstrated the best overall performance (AUC = 0.999, TSS = 0.984), indicating its exceptional accuracy and robustness. GBM and MAXENT also achieved similarly high predictive accuracy. ANN, CTA, MARS, XGBOOST, GLM, and FDA exhibited moderate performance, with most TSS values ranging from 0.74 to 0.78. SRE and GAM performed the worst, with TSS values of 0.563 and 0.554 respectively, both falling below the screening threshold (Table S1).

**FIGURE 2:**
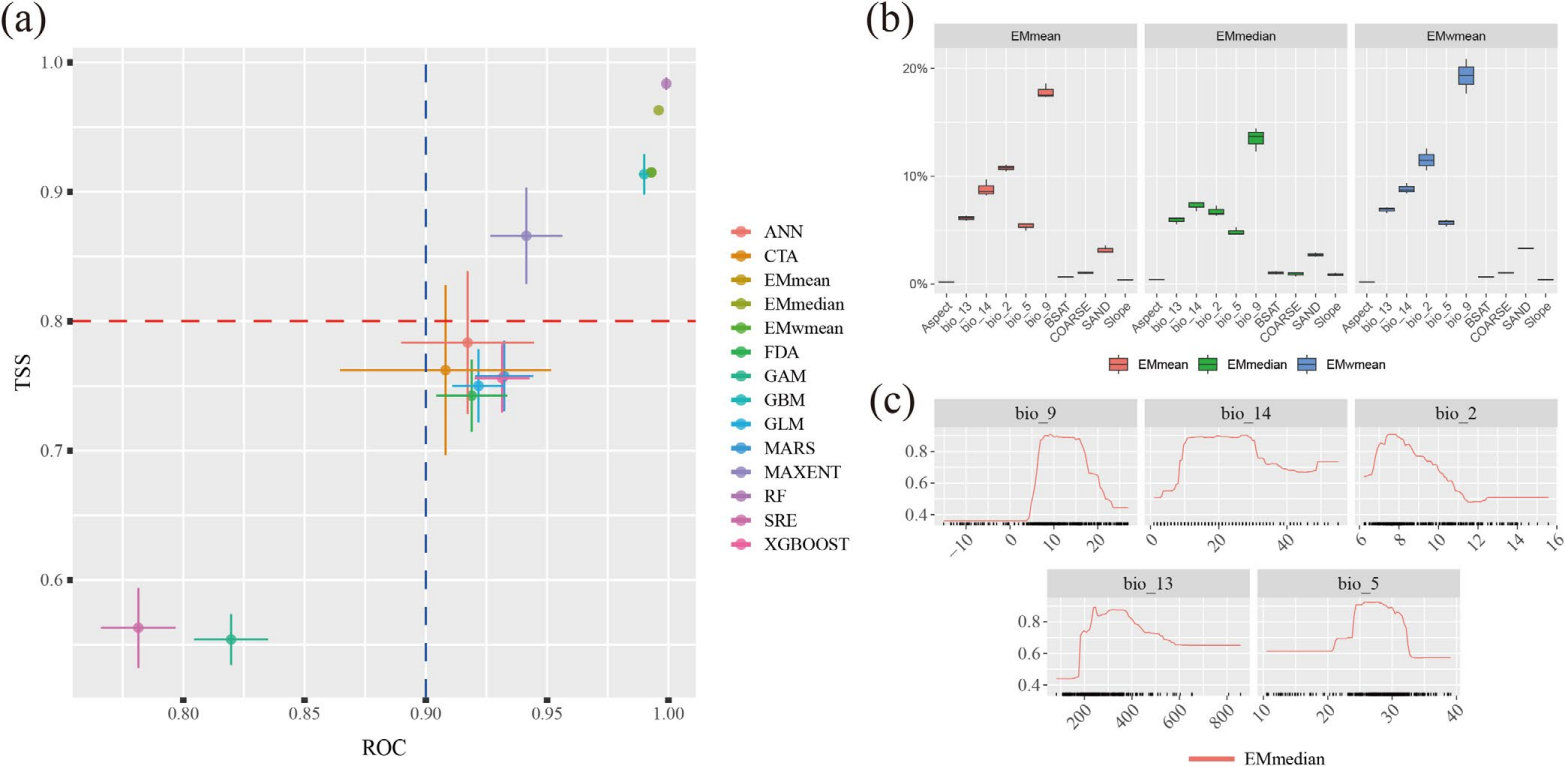
Model evaluation and variable contributions. (a) ROC and TSS values for all models. (b) Relative importance of environmental variables in ensemble models. (c) Response curves of environmental variables in EMmedian.

All ensembles demonstrated strong performance. EMmedian produced the most stable and reliable results (AUC = 0.996, TSS = 0.963), exhibiting higher accuracy and greater robustness compared with EMmean and EMwmean. The superior performance of EMmedian may stem from its inherent robustness against outliers, which effectively mitigates the bias introduced by individual anomalous models (Figure 2a). Therefore, we selected EMmedian as the final prediction model.

### 3.2 Key Environmental Factors

According to the variable importance results of the EMmedian model (Table 2), climatic variables are the dominant factors determining the spatial distribution of subgen. *Brachypetalum*. Among them, the mean temperature of the driest quarter (bio9) contributed the most, followed by precipitation of the driest month (bio14), mean diurnal temperature range (bio2), precipitation of the wettest month (bio13), and maximum temperature of the warmest month (bio5). In contrast, soil texture factors (SAND, BSAT, COARSE) and topographic factors (Slope, Aspect) each contributed less than 3%, and the variable importance results of EMmean and EMwmean were generally consistent (Figure 2b, Table S2).

**TABLE 2:** Relative importance of environmental variables in EMmedian.

Model	Variable	Importance (%)
EMmedian	bio9	13.49
EMmedian	bio14	7.27
EMmedian	bio2	6.73
EMmedian	bio13	5.94
EMmedian	bio5	4.85
EMmedian	SAND	2.72
EMmedian	BSAT	1.04
EMmedian	COARSE	0.94
EMmedian	Slope	0.89
EMmedian	Aspect	0.44

*Note:* The number retains two decimal places.

The analysis of environmental suitability ranges provided additional insight into the climatic tolerance thresholds of subgen. *Brachypetalum* (Table S3). Under general suitability conditions (habitat suitability ≥ 60%), subgen. *Brachypetalum* occurs within environments characterized by a mean diurnal temperature range (bio2) of 6.2°C–10.7°C, precipitation of the driest month (bio14) of 9–55 mm, and mean temperature of the driest quarter (bio9) of 6.1°C–20.1°C. Under high‐suitability conditions (habitat suitability ≥ 80%), the environmental requirements of subgen. *Brachypetalum* become more concentrated, representing its core ecological niche. The corresponding environmental thresholds are a mean diurnal temperature range (bio2) of 6.9°C–8.7°C, precipitation of the driest month (bio14) of 10–31 mm, and mean temperature of the driest quarter (bio9) of 7°C–16.7°C. Although soil and topographic factors showed some variation within the high‐suitability range (e.g., sand content 2.4%–34%, coarse fragment content 8.1%–36%), their overall constraining effects were far weaker than those of climatic factors (Figure 2c).

### 3.3 Current Distribution Pattern

Under the current climate scenario, the model projections show high consistency with field survey records, generally covering the known distribution range, with only slight overpredictions in some areas, mainly reflected in the expansion of medium and low suitability zones into southeastern South China (including the Nanling Mountains and Leizhou Peninsula) and the southwestern margin along the Sichuan Basin and the Three Gorges region. These overpredicted areas were mostly classified as low suitability zones, indicating some model extrapolation under marginal environmental conditions but with limited impact on the overall distribution pattern. Area statistics indicate that the total current suitable habitat covers approximately 395,371.74 km^2^, including 181,622.6 (46.0%) of low suitability, 125,266.87 km^2^ (31.7%) of moderate suitability, and 88,482.27 km^2^ (22.4%) of high suitability (Table 3).

**TABLE 3:** Potential suitable areas under different climate scenarios.

Climate scenarios	Low suitable (km^2^)	Medium suitable (km^2^)	Highly suitable (km^2^)	All suitable (km^2^)
LIG	6268.16	889.22	68.87	7226.25
MH	137,537.33	114,588.66	52,558.73	304,684.71
Current	181,622.6	125,266.87	88,482.27	395,371.74
2050_126	107,582.71	61,240.24	14,392.00	183,214.95
2050_245	145,184.51	79,294.27	13,344.99	237,823.77
2050_585	130,156.29	57,144.65	9278.32	196,579.26
2090_126	157,553.75	70,916.49	21,364.63	249,834.87
2090_245	131,401.22	46,369.72	3039.74	180,810.69
2090_585	53,094.23	6038.24	74.04	59,206.52

*Note:* The number retains two decimal places.

**FIGURE 3:**
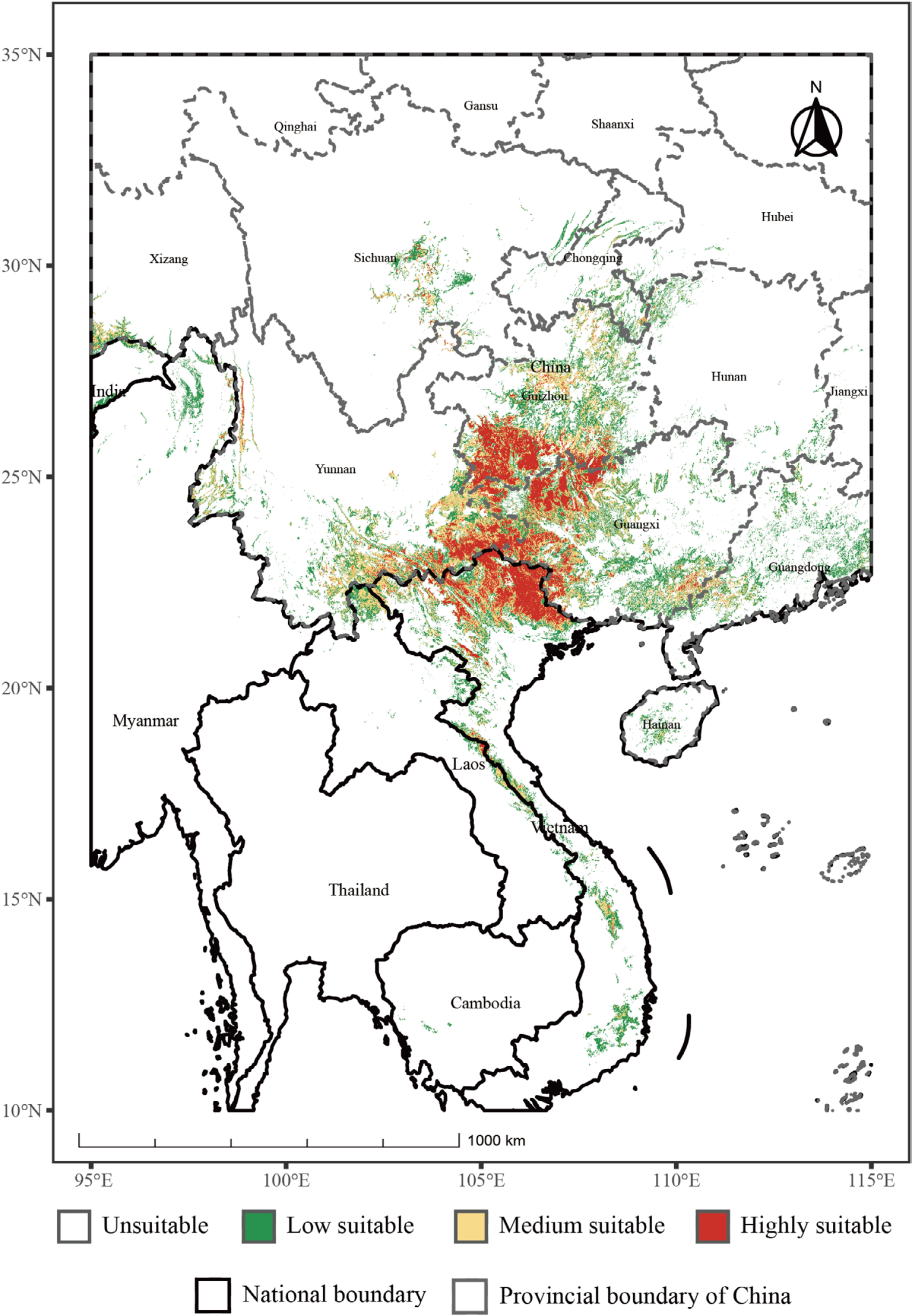
Potential suitable areas under the current climate condition.

Based on field occurrences, the high‐suitability zones delineate the core distribution range of subgen. *Brachypetalum*, concentrated mainly in three key regions: (1) southern Yunnan–southwestern Guangxi–northern Vietnam border; (2) southern Guizhou–northern Guangxi junction; and (3) southwestern Guizhou–eastern Yunnan mountainous area (Figure 3).

### 3.4 Dynamic Changes Under Climate Change

**FIGURE 4:**
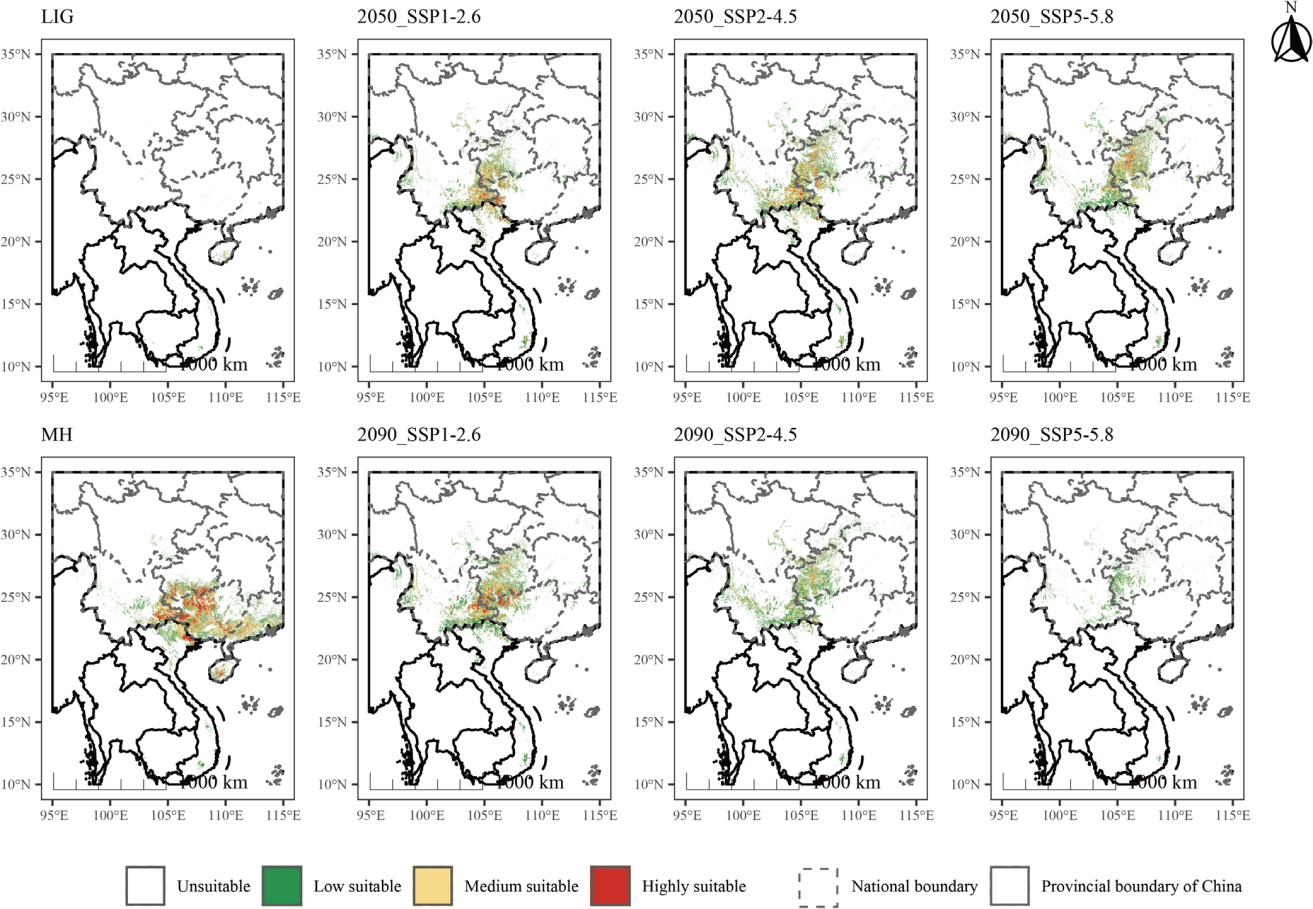
Potential suitable areas under paleoclimatic and future climate scenarios.

The potential suitable habitat area of subgen. *Brachypetalum* varied significantly across different climatic periods (Figure 4). In our results (Table 3), the present period had the largest extent of suitable habitat, whereas during LIG, the suitable area was extremely restricted (7226.25 km^2^ in total, high‐suitability areas are 6268.16 km^2^). During the MH period, the suitable habitat expanded substantially to 304,684.71 km^2^, and high‐suitability regions reached 52,558.73 km^2^.

**FIGURE 5:**
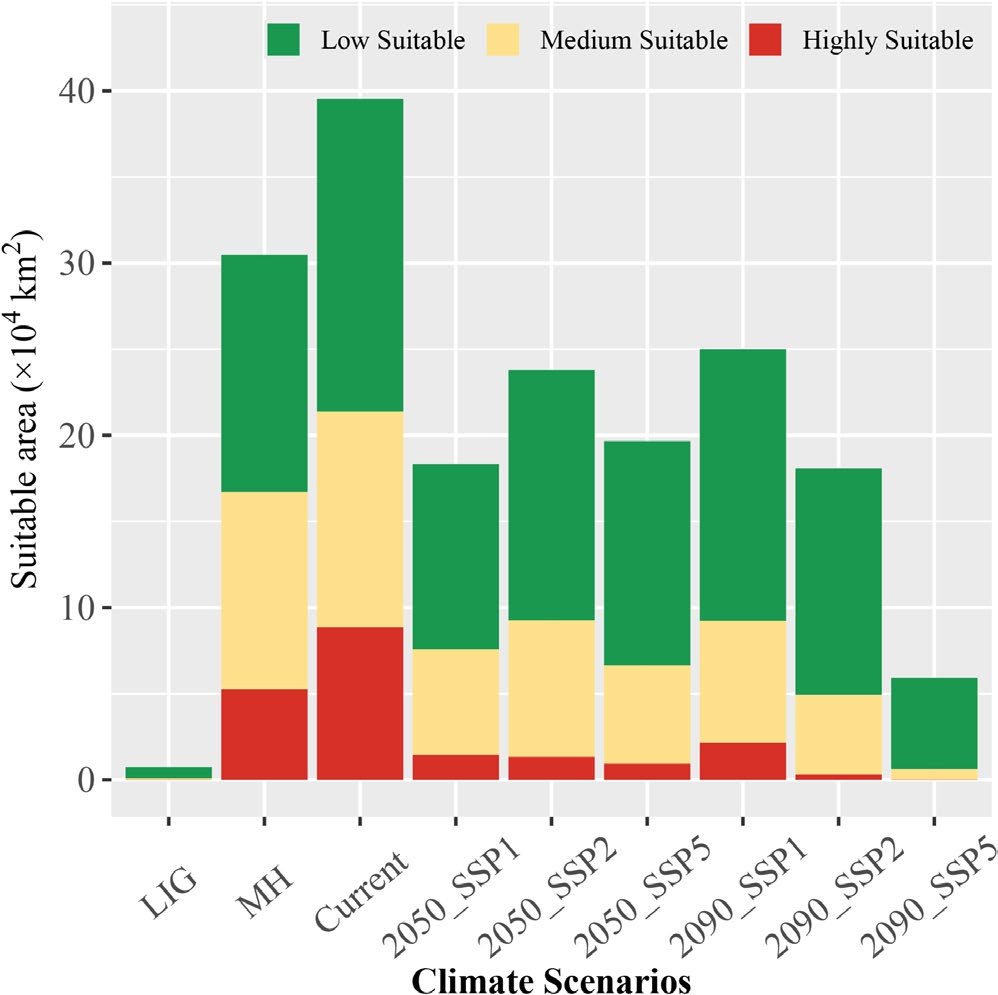
Changes in potential suitable area under different climate scenarios.

Under future climate scenarios, the total suitable habitat area is projected to decline overall and exhibit high instability. By 2050, distinct variation appeared among the emission scenarios: under the SSP2–4.5 scenario, the largest suitable area (237,823.77 km^2^) persisted, with high‐suitability zones remaining at 13,344.99 km^2^. By 2090, the changes in suitable habitat showed a dual trend. Under SSP1–2.6 scenario, the suitable area recovered to its optimal level for this stage, reaching 249,834.87 km^2^, with high‐suitability areas increasing to 21,364.63 km^2^. However, under SSP2–4.5 and SSP5–8.5 scenarios, suitable habitats sharply decreased, with high‐suitability areas under SSP5–8.5 scenario declining to very low levels (Figure 5).

**FIGURE 6:**
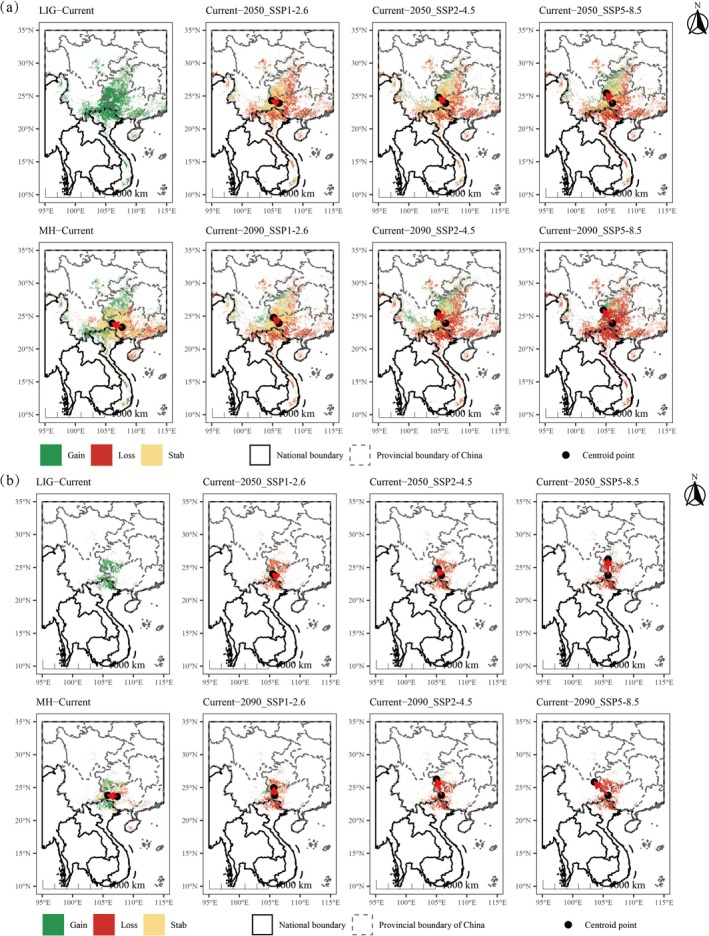
Range shifts and centroid migrations under different climate scenarios. (a) Changes in total suitable area compared with the current distribution and corresponding centroid migrations. (b) Changes in highly suitable area compared with the current distribution and corresponding centroid migrations.

Across multiple temporal periods, the suitable habitat of subgen. *Brachypetalum* experienced substantial changes, with the variation in highly suitable areas better reflecting its dynamic distributional trends compared with the overall suitable area (Figure 6). From MH to the present, the suitable habitat has expanded substantially. Under most future scenarios, habitat loss consistently exceeds habitat gain, particularly under high‐emission pathways, where both stable and newly gained suitable areas are markedly reduced (Table S4). Centroid analysis revealed that during the MH period, the distribution was centered in central–western Guangxi, China, whereas at present, the centroid has shifted to the central region of the southwestern China karst plateau (Guizhou–Guangxi–Yunnan junction), indicating a distinct westward migration of suitable habitats. Under future climate scenarios, both total and highly suitable habitat centroids of subgen. *Brachypetalum* exhibit a consistent pattern, showing a tendency to shift northward and northwestward across all scenarios (Figure 6).

### 3.5 MESS and MoD Results

**FIGURE 7:**
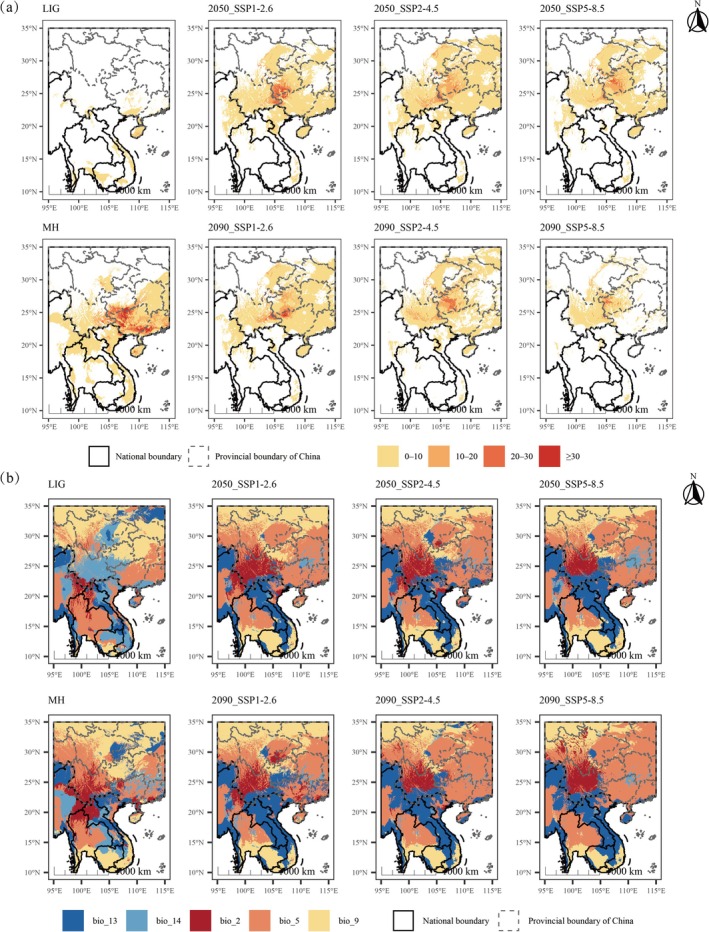
MESS and MoD under different climate scenarios. (a) Multivariate environmental similarity surface (MESS) analysis showing areas with environmental conditions outside the calibration range (negative values). (b) Most dissimilar variable (MoD) analysis identifying the climatic variables that contribute most to environmental dissimilarity under future scenarios.

MESS analysis results indicate that environmental similarity in suitable habitats for the subgen. *Brachypetalum* varies significantly across different climate scenarios (Figure 7a). In the LIG period, most areas exhibited negative MESS values, signifying that climatic conditions largely exceeded the range of the training dataset, which corresponds to the sharply reduced suitable range seen in the projections. During the MH period, southern Guizhou and northern Guangxi exhibited substantial environmental dissimilarity, with more than 30% of the region presenting high extrapolation risk. In contrast, under current and future climate scenarios, the environmental conditions of suitable habitats remained largely similar to those of the training dataset, with more than 85% of the area having MESS values between 0 and 10, and less than 2% of the area with MESS > 30.

The MoD analysis further revealed the key environmental variables driving dissimilarity among scenarios. In LIG and MH periods, all environmental variables exhibited large differences, with no clear dominant factor, suggesting a systematic difference between past and present climatic patterns. Under future scenarios, temperature‐related variables dominated climate change across the entire study area, while humidity‐related factors played a more significant role within the core distribution regions of subgen. *Brachypetalum* (Figure 7b).

### 3.6 Evaluation of Protected Area Effectiveness

In the current climate scenario, approximately 4220.85 km^2^ of highly suitable habitat lies within existing protected areas, accounting for a substantial proportion of the present core distribution zone. Under future scenarios, this coverage declines markedly. By 2050, only 547.50 km^2^ (12.97%), 459.86 km^2^ (10.89%), and 202.42 km^2^ (4.80%) remain under SSP1–2.6, SSP2–4.5, and SSP5–8.5 scenarios, respectively. By 2090, coverage slightly recovers under the low‐emission scenario (915.66 km^2^, 21.69%), but declines to 168.95 km^2^ (4.00%) and less than 10 km^2^ (0.2%) under the medium‐ and high‐emission scenarios, indicating a substantial reduction in protection effectiveness.

Comparison of protected area coverage across future climate scenarios shows that current protected areas provide limited coverage of newly formed suitable habitats, leaving many potential areas outside the existing protection network. As presented in Table 4, habitat changes are strongly asymmetric, with loss consistently exceeding gains across all scenarios. For example, in 2050 under SSP1–2.6, highly suitable habitats lost 3811.18 km^2^ (90.29%) while gaining only 166.99 km^2^ (3.96%). Under SSP2–4.5 and SSP5–8.5 scenarios, loss further increased to 3915.87 km^2^ (92.77%) and 4123.89 km^2^ (97.70%), whereas gains remained limited (4.37% and 2.77%, respectively).

**TABLE 4:** Range shifts of protected highly suitable habitats under different climate scenarios.

Climate scenarios change	Loss area (km^2^/ percentage)	Stab area (km^2^/ percentage)	Gain area (km^2^/percentage)
Current‐2050_126	3811.18/90.29%	409.67/9.71%	166.99/3.96%
Current‐2050_245	3915.87/92.77%	304.98/7.23%	184.66/4.37%
Current‐2050_585	4123.89/97.7%	96.96/2.3%	117.08/2.77%
Current‐2090_126	3598.73/97.7%	622.12/14.74%	321.56/7.62%
Current‐2090_245	4180.5/85.26%	40.35/0.96%	134.75/3.19%
Current‐2090_585	4220.85/99.04%	0/0%	8.53/0.2%

*Note:* The number retains two decimal places.

By 2090, this imbalance becomes even more pronounced. Under SSP1–2.6, although 622.12 km^2^ (14.74%) of habitat remains stable, losses still reach 3598.73 km^2^ (97.70%). In contrast, under SSP5–8.5, habitat loss rises to 4220.85 km^2^ (99.04%), with negligible gains (0.20%) and no stable habitat remaining, indicating extremely limited buffering capacity under extreme climate conditions (Table 4).

## 4 Discussion

### 4.1 Model Performance and Applicability

Although different algorithmic models can provide several numerical evaluation metrics, the applicability of the model is often more critical than mere accuracy when predicting the distribution of rare and endangered taxa (Hellegers et al. 2025). Subgen. *Brachypetalum* occurs in highly fragmented karst habitats with narrow ecological niches, which increases the risk of model overfitting. To better reflect these distribution characteristics, the disk‐based pseudo‐absence strategy was adopted, as it constrains background sampling to ecologically relevant areas. This approach helps reduce overfitting and improves the ecological realism of model predictions.

Regarding algorithm selection, ensemble models demonstrate superior generalization capabilities compared with single models, particularly when dealing with limited sample sizes (Breiner et al. 2015). They effectively mitigate the uncertainty arising from sampling sparsity. For this study, compared with RF, EMmedian better balances the strengths and limitations of different algorithms, avoiding bias caused by individual models over‐relying on specific variable types.

Moreover, the strong spatial heterogeneity of suitable habitats emphasizes that binary classification of habitat suitability may oversimplify ecological reality. For species with highly insular and fragmented distributions, distinguishing among different levels of habitat suitability provides a more realistic representation of habitat quality and ecological gradients (Hellegers et al. 2025). In particular, highly suitable areas likely correspond to core habitats that sustain stable populations, making them especially critical for conservation planning and priority setting.

### 4.2 Identification of Key Climatic Drivers

The dominance of climatic factors in shaping the distribution of subgen. *Brachypetalum* is consistent with observations from other *Paphiopedilum* taxa (Dutta and De 2022). Mean temperature in the driest quarter (bio9) and precipitation in the driest month (bio14) were identified as the primary limiting factors, highlighting the critical role of dry‐season hydrothermal conditions. This pattern reflects the strong dependence of subgen. *Brachypetalum* on stable dry‐season environments, which is consistent with its restricted distribution in karst regions. For terrestrial orchids with shallow root systems and high water requirements, excessive deviation of dry‐season conditions from the optimal range not only compromises plant survival but also determines seed germination and reproductive success. This readily leads to reduced individual survival rates and even local extinction (Xian et al. 2025).

Topography and edaphic properties act as secondary filters beneath the dominant climatic constraints. For example, slope and aspect modulate microclimatic variation such as moisture and light, whereas soil texture (e.g., sand content, proportion of coarse fragments) influences drainage and nutrient dynamics. Subgen. *Brachypetalum* are associated with exposed karst habitats, where shallow soils and rocky substrates impose strong ecological filtering. And, the absence of endosperm in seeds makes successful germination dependent on symbiotic mycorrhizal fungi, further constraining establishment (Tian et al. 2024). Human activities, including land‐use change and local community development, may also influence species persistence and dispersal (Zhou et al. 2021). Although these factors may determine individual survival at micro‐scales, their spatial heterogeneity is far less than that of climatic variables at regional or global scales, making them less likely to define broad distribution boundaries.

In other words, climate determines a species’ “fundamental niche,” whereas nonclimatic factors mainly affect the “realized niche,” influencing local distribution details rather than overall range patterns.

### 4.3 Patterns of Suitable Habitat Change

The study revealed the distributional dynamics and migration patterns of subgen. *Brachypetalum* under historical, current, and future climatic scenarios. During LIG period (ca. 129–117 ka BP, Shackleton et al. 2020), East and Southeast Asia experienced generally higher temperatures, along with intensified East Asian monsoons and enhanced monsoonal rainfall. Concurrent regional aridity and sea‐level rise (6–9 m above modern levels) submerged parts of the lowlands in South China and Indochina, leading to severely fragmented suitable habitats. As a result, the suitable habitats of subgen. *Brachypetalum* during this time were minimal and spatially discontinuous, maintained primarily within a few refugial zones influenced by climate and sea‐level constraints. In contrast, MH (~6000 years BP, Steig 1999) is regarded as the “optimum period” of Holocene climate. At that time, the East Asian summer monsoon was strong and stable, with markedly increased precipitation across eastern China, South China, and the Indochina Peninsula, and temperatures similar to or slightly higher than the present, yielding optimal hydrothermal conditions overall. Our results show that during the MH period, the suitable habitats of subgen. *Brachypetalum* expanded considerably, forming a broad and continuous distribution pattern. This suggests that the strong and stable monsoon system provided favorable ecological conditions and offers new spatial evidence supporting phylogenetic and biogeographical hypotheses of *Paphiopedilum* taxa migrating northward from tropical regions. This historical evolutionary trajectory aligns with other phylogenetic and biogeographical findings, and our study provides compelling evidence supporting the hypothesis that the *Paphiopedilum* originated in mainland Southeast Asia and the Sunda region (Górniak et al. 2025).

Under future climate scenarios, the total area of suitable habitat for subgen. *Brachypetalum* is projected to shrink significantly while exhibiting a pronounced northward shift. Suitable habitat change exhibits a clear gradient across emission scenarios: under low‐emission conditions, climatic changes are relatively limited, resulting in moderate habitat loss and only minor northward shifts of the core distribution; in contrast, under high‐emission scenarios, habitat contraction becomes pronounced, accompanied by a greater northward displacement of the core distribution. Overall, the distribution centroid consistently shifts northwestward, gradually converging toward the Yunnan–Guizhou Plateau, with Guizhou projected to become a central area of concentration. The persistence of suitable habitats in southwestern Guizhou suggests the presence of potential climate refugia, likely associated with the highly heterogeneous karst mountainous landscape, which can buffer climatic variability and create microrefugia that enhance habitat stability (Tang et al. 2018). This pattern aligns with plants' universal adaptive response of “migrating toward cooler, wetter environments” in response to warming, reaffirming the dominant role of climatic factors in determining distribution dynamics (Wang, Li, et al. 2025; McLaughlin et al. 2017).

MESS and MoD analyses provide additional support for the uncertainty in potential distribution areas across different periods. MESS values were predominantly negative during the LIG period, indicating environmental conditions beyond the range of modern training data, which explains the limited and highly fragmented predicted distribution for this period. In contrast, future projections show that environmental variability in highly suitable areas remains relatively low (MESS < 10). Simultaneously, MoD analysis reveals significant variation in key climatic variables, particularly in southern Guizhou and northern Guangxi. It should be noted that this pattern reflects differences in environmental dissimilarity rather than variable importance in determining species distribution. Both results indicate that the distribution dynamics of subgen. *Brachypetalum* are influenced by multiple environmental drivers, even though certain variables play dominant roles in the ensemble models.

### 4.4 Conservation and Recommendations

As a representative case, in recent decades, subgen. *Brachypetalum* has garnered significant attention from all sectors of society and achieved positive results in conservation practices. Direct interventions targeting human digging and illegal trade have significantly decreased (Zhou et al. 2021). But the conservation of subgen. *Brachypetalum* still remains challenging due to its strong dependence on highly specific habitats and sensitivity to environmental disturbance. Although existing protected areas cover part of the current highly suitable habitats, our results reveal substantial conservation gaps under future climate scenarios, particularly in newly emerging and stable highly suitable areas. This indicates that the current protected area network is insufficient to accommodate ongoing shifts in habitat suitability.

Based on the projected spatial dynamics, conservation priorities should focus on areas that combine high suitability, long‐term stability, and landscape connectivity. In particular, the Yunnan–Guizhou–Guangxi junction, centered on southwestern Guizhou, is identified as a key region where suitable habitats persist and converge under multiple scenarios. This region likely functions as both a climate refugium and a future distribution core, making it critical for long‐term conservation planning.

To address these challenges, targeted conservation actions should be implemented, including (1) expanding existing protected areas to incorporate stable and newly suitable habitats, (2) establishing microreserves for small and isolated species populations, (3) enhancing ecological restoration in degraded habitats to improve connectivity, and (4) strengthening long‐term monitoring and adaptive management to respond to climate‐driven distribution shifts. In addition, cross‐regional conservation coordination should be promoted to facilitate species migration along the projected northwestward shift of suitable habitats.

## 5 Conclusions

The current suitable habitats of subgen. *Brachypetalum* are concentrated in three core regions: the southern margin of the Yunnan–Guizhou Plateau to the northern Indochina border zone, the Guizhou–Guangxi junction, and the Yunnan–Guizhou junction, exhibiting a typical “widespread but rare species” aggregation pattern. Its spatial distribution is mainly governed by the mean temperature of the driest quarter (bio9) and precipitation of the driest month (bio14).

Temporally, it has undergone a dynamic process of “LIG extreme contraction – MH significant expansion – Current peak – Future decline.” Under the overarching trend of climate warming, the core suitable habitats exhibit high sensitivity to climatic changes, particularly under the 2090 SSP5–8.5 scenario, where highly suitable areas are projected to decline to very low levels (75.85%–99.92% reduction). Centroid migration results indicate that under future scenarios, the distribution center consistently shifts north–northwestward, gradually moving toward the Guizhou mountainous area, reflecting a “cooler and wetter” adaptive response under warming conditions.

Protected area overlay analysis indicates that while existing reserves cover parts of the present core habitats, protection of newly projected high‐suitability regions is highly insufficient, with major gaps concentrated in the Yunnan–Guizhou–Guangxi border zone. With continued warming driving habitat shrinkage and range shifts, the subgenus will face compounded threats of habitat loss and localized extinction if no targeted conservation measures are implemented. Therefore, we recommend prioritizing conservation in stable and future high‐suitability areas, particularly in southwestern Guizhou and along the Yunnan–Guizhou–Guangxi junction, by expanding existing reserves, establishing conservation nodes, and enhancing ecological connectivity. Strengthening cross‐border cooperation and ecological corridor connectivity will be crucial for maintaining habitat stability and ensuring the long‐term survival of subgen. *Brachypetalum* under future climate change.

## Author Contributions

**Hao Zhang:** conceptualization (lead), data curation (lead), methodology (lead), software (lead), visualization (lead), writing – original draft (lead), writing – review and editing (lead). **Mingtai An:** conceptualization (equal), funding acquisition (lead), project administration (lead), supervision (lead), writing – review and editing (equal). **Jianghong Yu:** data curation (equal), supervision (equal), validation (lead), visualization (supporting), writing – review and editing (supporting).

## Funding

This work was supported by the National Natural Science Foundation of China, 32360101, Guizhou Province Wild Orchid Monitoring (Phase II), dzwz202510, and Survey and Assessment of Newly Added National Key Protected Wild Plant Resources in Guizhou Province (Phase III), MCHC‐ZD20242057, Guizhou Provincial Basic Research Program (Natural Science), QKHJC‐ZK[2023]general235.

## Conflicts of Interest

The authors declare no conflicts of interest.

## Supporting information


**Table S1:** ROC and TSS values for all models.
**Table S2:** Relative importance of environmental variables in ensemble models.
**Table S3:** Response curves of environmental variables in EMmedian.
**Table S4:** Range shifts and centroid migrations under different climate scenarios.

## Data Availability

All necessary data have already been provided in the main text and Supporting Information.
